# The Role of the Pentose Phosphate Pathway in Diabetes and Cancer

**DOI:** 10.3389/fendo.2020.00365

**Published:** 2020-06-09

**Authors:** Tongxin Ge, Jiawen Yang, Shihui Zhou, Yuchen Wang, Yakui Li, Xuemei Tong

**Affiliations:** Department of Biochemistry and Molecular Cell Biology, Shanghai Key Laboratory for Tumor Microenvironment and Inflammation, Key Laboratory of Cell Differentiation and Apoptosis of Chinese Ministry of Education, Shanghai Jiao Tong University School of Medicine, Shanghai, China

**Keywords:** the pentose phosphate pathway, metabolism, obesity, diabetes, cancer

## Abstract

The pentose phosphate pathway (PPP) branches from glucose 6-phosphate (G6P), produces NADPH and ribose 5-phosphate (R5P), and shunts carbons back to the glycolytic or gluconeogenic pathway. The PPP has been demonstrated to be a major regulator for cellular reduction-oxidation (redox) homeostasis and biosynthesis. Enzymes in the PPP are reported to play important roles in many human diseases. In this review, we will discuss the role of the PPP in type 2 diabetes and cancer.

## Introduction

The pentose phosphate pathway (PPP), also known as the pentose phosphate shunt, is an important part of glucose metabolism. The PPP branches after the first step of glycolysis and consumes the intermediate glucose 6-phosphate (G6P) to generate fructose 6-phosphate (F6P) and glyceraldehyde 3-phosphate (G3P) through the oxidative and non-oxidative branches of the PPP. Unlike glycolysis and glucose aerobic oxidation, the PPP does not provide adenosine 5′-triphosphate (ATP) to meet the energy demands of cells. Instead, it supplies NADPH and ribose 5-phosphate (R5P). These two metabolites are vital for the survival and proliferation of cells. R5P is a building block for nucleic acid synthesis. NADPH is the reducing power required for the synthesis of fatty acids, sterols, nucleotides and non-essential amino acids (1, 2). Moreover, NADPH-derived conversion of oxidized glutathione (GSSG) to reduced glutathione (GSH) *via* glutathione reductase is important for cellular antioxidant defenses. Interestingly, NADPH also serves as the substrate of NADPH oxidases (NOXs) which produce reactive oxygen species (ROS) (3).

**Figure 1:**
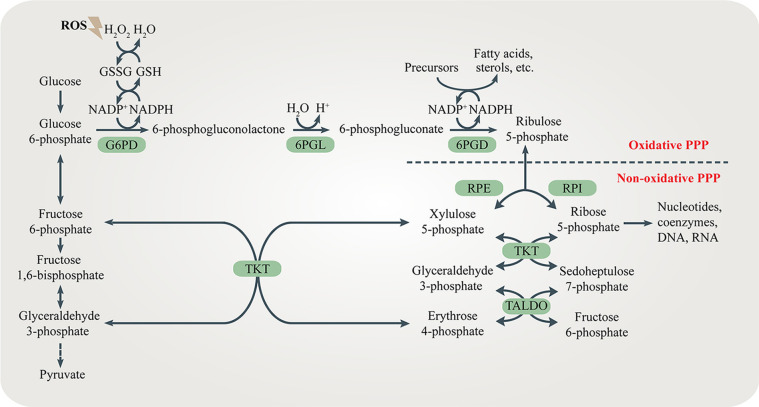
The pentose phosphate pathway (PPP). The PPP branches after the first step of glycolysis and goes back to fructose 6-phosphate and glyceraldehyde 3-phosphate in the glycolytic and gluconeogenic pathway. The PPP produces R5P and NADPH for biosynthesis and redox regulation. Enzymes in the oxidative and non-oxidative PPP are shaded in green.

Both the oxidative branch and non-oxidative branch of the PPP take place in the cytosol (Figure 1). Glucose 6-phosphate dehydrogenase (G6PD) is the rate-limiting enzyme of the oxidative PPP, determining the flux of G6P directed into the pathway. G6PD catalyzes the conversion of G6P to 6-phosphogluconolactone, accompanied by NADPH production. 6-phosphogluconolactonase (6PGL) is the enzyme that hydrolyses 6-phosphogluconolactone to produce 6-phosphogluconate (6PG). 6-phosphogluconate dehydrogenase (6PGD) converts 6-PG to ribulose 5-phosphate (Ru5P) and generates NAPDH (Figure 1). The largest contributor to cytosolic NADPH is the oxidative PPP in mammalian cells. Moreover, at least 3 other cytoplasmic enzymes including isocitrate dehydrogenase 1 (IDH1), malic enzyme 1 (ME1) and 10-formyltetrahydrofolate dehydrogenase (ALDH1L1) contribute to NADPH synthesis in cytosol. Furthermore, mitochondrial NADPH production is dependent on at least 5 mitochondrial enzymes including nicotinamide nucleotide transhydrogenase (NNT), isocitrate dehydrogenase 2 (IDH2), malic enzyme 3 (ME3), mitochondrial homolog of 10-formyltetrahydrofolate dehydrogenase (ALDH1L2) and methylenetetrahydrofolate dehydrogenase 1 like (MTHFD1L) (4).

The non-oxidative branch is composed of a series of reversible transfer reactions of chemical groups. Ribose 5-phosphate isomerase (RPI) and ribulose 5-phosphate epimerase (RPE) catalyze reversible reactions converting Ru5P to R5P and xylulose 5-phosphate (Xu5P), respectively. TKT catalyzes two reversible reactions. One is the conversion of Xu5P and R5P to G3P and sedoheptulose 7-phosphate (S7P). The other is the conversion of Xu5P and erythrose 4-phosphate (E4P) to G3P and F6P. Therefore, TKT can bi-directionally regulate the carbon flux between the non-oxidative PPP and glycolysis or gluconeogenesis. Transaldolase (TALDO) reversibly converts G3P and S7P to E4P and F6P. The non-oxidative branch not only replenishes metabolites of the oxidative branch by the reversible reactions, but also regulates the flux of glycolysis or gluconeogenesis by providing F6P and G3P (5) (Figure 1).

## The Role of the PPP in Type 2 Diabetes Mellitus (T2DM)

T2DM is a chronic metabolic disease featured by persistently abnormal hyperglycemia, which can cause serious chronic damage to kidneys, eyes, and nerves. Deregulated insulin secretion and progressive insulin resistance are two main characteristics of T2DM (6). Over the past few decades, studies on the pathogenesis of T2DM have revealed a close relationship between the PPP, obesity-related insulin resistance and T2DM. In this part, we will mainly focus on the role of the PPP in obesity-related insulin resistance, insulin secretion and chronic diabetic complications.

### The Role of the PPP in Obesity-Related Insulin Resistance

The term “insulin resistance” indicates that insulin-responsive tissues such as the liver, adipose tissue, and skeletal muscle reduce insulin-mediated glucose uptake, contributing to hyperglycemia (7). Pancreatic islet β cells, therefore, have to secrete more insulin to compensate for insulin resistance, resulting in hyperinsulinemia which leads to dysfunction of β cells and T2DM.

Obesity is closely related to the onset of insulin resistance. Chronic obesity-induced inflammation is one of the major causes of obesity-related insulin resistance. Adipose tissue macrophages (ATMs) surrounding dead adipocytes cause obesity-induced inflammation and secrete pro-inflammatory cytokines leading to local insulin resistance in adipose tissues (8). The severity of obesity-induced inflammation correlates with the degree of obesity. Abnormally increased number and activity of ATMs as well as higher ratio of pro-inflammatory to anti-inflammatory macrophages are both hallmarks of obesity-induced inflammation (9). Different activity of the oxidative PPP in macrophages contributes to the functional discrepancy of macrophages. Pro-inflammatory M1 macrophages show enhanced glycolysis and PPP flux which provides more energy and NADPH to trigger inflammatory responses, secrete pro-inflammatory cytokines and recruit more immune cells. However, anti-inflammatory M2 macrophages, displaying decreased glycolysis and PPP flux, work adversely to resolute inflammatory responses, secreting anti-inflammatory cytokines to inhibit M1 macrophages (10). The pro-inflammatory cytokines released by M1 macrophages include tumor necrosis factor-α (TNF-α) and interleukin 1β (IL-1β). TNF-α and IL-1β contribute to insulin resistance in adipose tissues by altering the insulin receptor signaling pathway *via* the stress-responsive c-Jun-NH2-terminal kinase (JNK 1/2), inhibitor of κB kinase (IKK) and mitogen-activated protein kinase (MAPK) p38 (11, 12). In contrast, M2 macrophages secret the anti-inflammatory cytokines including interleukin 10 (IL-10) and arginase1 and maintain insulin sensitivity (12, 13). In addition, pro-inflammatory cytokines recruit more monocytes to adipose tissues, resulting in more severe obesity-induced inflammation. This amplifying feedback loop aggravates local insulin resistance.

Moreover, obesity-induced inflammation can lead to insulin resistance in the skeletal muscle or liver, resulting in systemic insulin resistance (9, 14, 15). Pro-inflammatory cytokines promote lipolysis in adipocytes, leading to elevated levels of circulating free fatty acids (FFAs). In the skeletal muscle, FFAs reduce insulin-stimulated glucose intake, resulting in skeletal muscle insulin resistance (16). On the other hand, lipolysis in adipose tissues increases hepatic acetyl CoA levels and pyruvate carboxylase activity which further promotes hepatic glucose production. Both hepatic glucose production and pro-inflammatory cytokines contribute to hepatic insulin resistance (8, 17, 18). FFAs increase production of ROS in liver, leading to severe insulin resistance and nonalcoholic steatohepatitis (NASH) (19). Furthermore, long-term exposure to elevated levels of FFAs contributes to pancreatic β cells dysfunction and death (20, 21).

Recent studies suggest that the PPP might serve as a novel and promising target for modulating obesity-induced inflammation and insulin sensitivity in different tissues. Over-nutrition causes excessive FFAs released from adipose tissues which up-regulates G6PD expression in ATMs. Elevated levels of G6PD in ATMs can cause obesity-related inflammation (22). Pro-inflammatory cytokines also increase G6PD expression in adipocytes. Accordingly, adipocytes secrete adipocytokines including resistin and TNF-α which further stimulate inflammatory responses and recruit more monocytes to the inflamed adipose tissues (23). This vicious cycle promotes obesity-related insulin resistance, resulting in severe T2DM. The non-oxidative PPP in adipose tissues also plays an important role in regulating insulin sensitivity. TKT deficiency in adipocytes results in R5P accumulation and reduced glycolysis, accompanied by increased lipolysis and fatty acid β-oxidation. Therefore, loss of TKT in adipose tissues alleviates high fat diet (HFD)-induced obesity, leading to reduced hepatic steatosis and improved insulin sensitivity (24). Moreover, increased expression of G6PD in hepatocytes generates more NADPH for *de novo* lipogenesis (DNL) which promotes hepatic steatosis and insulin resistance (25, 26). Interestingly, excessive NADPH in liver may contribute to oxidative stress *via* NOXs, leading to liver damage and insulin resistance (27). The peroxisome proliferator-activated receptor δ (PPARδ) reduces hepatic glucose output and improves insulin sensitivity partly by regulating the PPP flux (28). Furthermore, G6PD in skeletal muscle regulates glucose uptake and insulin sensitivity (29). As an excellent intervention in metabolic diseases, exercise increases the level of peroxisome proliferator-activated receptor γ coactivator 1α (PGC-1α) which promotes G6PD transcription and higher intramyocellular lipid (IMCL) content in skeletal muscles. The combination of increased muscle PGC-1α expression and exercise greatly enhances insulin sensitivity (30, 31).

**Figure 2:**
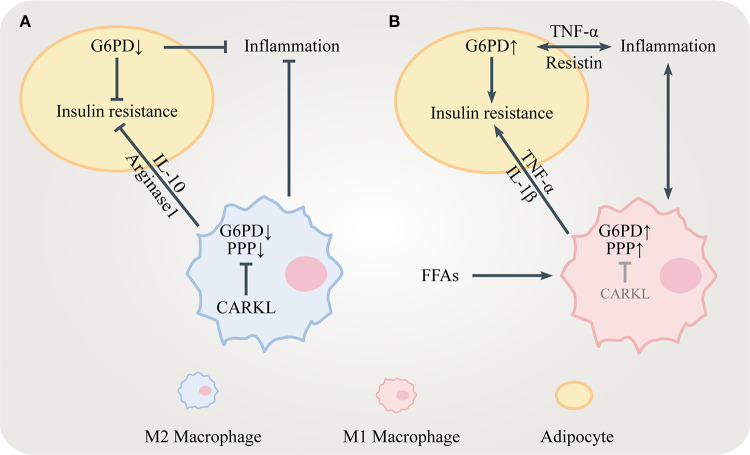
The role of the PPP in insulin resistance. **(A)** CARKL is highly expressed in M2 macrophages, limiting the PPP flux by inhibiting G6PD. M2 macrophages release anti-inflammatory mediators including IL-10 and arginase 1 to maintain insulin sensitivity. Decreased G6PD in adipocytes suppresses inflammation and ameliorates insulin resistance. **(B)** FFAs and pro-inflammatory cytokines including TNF-α, IL-1β, and resistin increase G6PD expression in both adipocytes and M1 macrophages, which stimulate inflammatory responses leading to insulin resistance.

Carbohydrate kinase-like protein (CARKL), also known as sedoheptulokinase (SHPK), is a carbohydrate kinase catalyzing the phosphorylation of sedoheptulose to S7P (32). Since S7P is the substrate for TKT and TALDO, CARKL is important for regulating the flux through the PPP. High level of S7P generated by CARKL restricts the reversible reaction in the non-oxidative PPP, limiting the flux through the PPP. CARKL is highly expressed in M2 macrophages and its down-regulation is critical for proper M1 polarization (33, 34) (Figure 2). It is reasonable to assume that regulating CARKL may reduce obesity-induced inflammation, leading to increased insulin sensitivity. Furthermore, whether TKT and TALDO can direct macrophage polarization should be studied in the future.

### The Role of the PPP in Insulin Secretion

Insulin is stored in granules and released *via* exocytosis from pancreatic islet β cells in response to glucose in a biphasic manner, which is known as the triggering pathway and the amplifying pathway (35). The amplifying pathway accounts for the majority of glucose-stimulated insulin secretion (GSIS). Decreased insulin secretion in the amplifying pathway is often observed in patients with T2DM. Therefore, how to simulate insulin secretion during the amplifying pathway is important for the prevention and treatment of T2DM.

**Figure 3:**
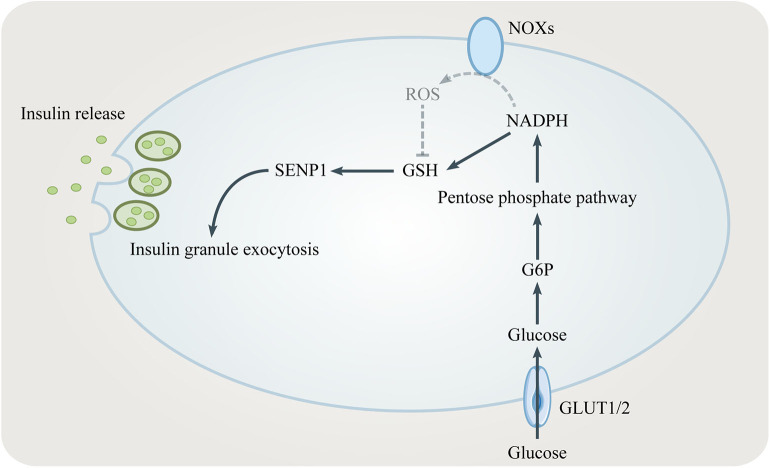
The role of the PPP in insulin secretion. NADPH from the PPP converts oxidized glutathione (GSSG) to reduced glutathione (GSH). GSH elicits insulin granule exocytosis *via* SENP1. However, NADPH might inhibit insulin secretion by promoting NADPH oxidases (NOXs).

NADPH is one key modulator of the amplifying pathway because it converts GSSG to GSH which elicits insulin granule exocytosis *via* sentrin/SUMO-specific protease-1 (SENP1) (35, 36) (Figure 3). Being a major source for NADPH, the PPP regulates the GSIS-related NADPH/GSH/SENP1 pathway. Only optimal levels of G6PD and 6PGD, two enzymes generating NADPH in the PPP, are beneficial to GSIS. Patients with G6PD deficiency show decreased insulin secretion (37). Inhibition of G6PD and 6PGD not only blocks GSIS but also increases oxidative stress and β cells apoptosis (38, 39). However, overexpression of G6PD also negatively influences GSIS which is due to increased expression of NADPH oxidases (NOXs) and ROS accumulation (40, 41). In conclusion, the PPP/NADPH/GSH/SENP1 pathway needs to be precisely controlled to achieve beneficial GSIS.

### The Role of the PPP in Chronic Diabetic Complications

Diabetes can lead to diabetic nephropathy, diabetic retinopathy, diabetic cardiomyopathy, diabetic macroangiopathy and other chronic complications. Oxidative stress can cause these complications by activating the hexosamine pathway, the advanced glycation end products (AGEs) pathway and the diacylglycerol (DAG)-protein kinase C (PKC) pathway (42).

Hyperglycemia decreases G6PD activity through the activation of protein kinase A (PKA) and increase of intracellular oxidative stress, leading to chronic kidney injury, and diabetic kidney disease (DKD) (43–45). Moreover, overexpression of G6PD in endothelial cells prevents diabetic cardiomyopathy by decreasing ROS accumulation and increasing endothelial cell viability (46).

TKT plays an important role in preventing hyperglycemia-induced vascular cell dysfunction (47). The cofactor of TKT is thiamine diphosphate (TDP), the active form of thiamine. Thiamine deficiency and decreased TKT activity has been reported to contribute to diabetic complications (48). Low plasma thiamine was found in patients with DKD and diabetic rats. After high-dose thiamine therapy, the progression of proteinuria and microalbuminuria was reversed in both patients and animal models, indicating that regulating the activity of TKT may be a promising therapy in treating DKD (47, 49, 50). Benfotiamine, a lipid-soluble thiamine derivative, can prevent diabetic retinopathy and cardiomyopathy as well as accelerate the healing of diabetic limbs by activating TKT (51–53). However, benfotiamine did not show promising efficacy in phase II and IV trials for the treatment of DKD or diabetic peripheral nerve function (54, 55). Therefore, other transketolase activators await further investigation.

## The Role of the PPP in Cancer

The PPP is critical for cancer prevention and treatment because NADPH and R5P play important roles in regulating DNA damage response, metabolism, and proliferation in cancer cells. Various enzymes in the PPP have been shown to be potential targets in cancer therapy. These proteins not only function as metabolic enzymes, but also participate in the regulation of other cellular activities. Therefore, we will summarize recent findings in upstream signaling pathways regulating PPP enzymes in cancer initiation and progression (Table 1).

**Table 1:** The regulation of PPP enzymes in cancer cells.

**Enzyme**	**Cancer type**	**Regulation**	**References**
G6PD	Lung cancer	TAp73 transcriptionally activates *G6PD*	(56, 57)
		Nrf2 transcriptionally activates *G6PD*	(58)
		O-GlcNAcylation of G6PD at S84 enhances its activity	(59)
	Liver cancer	ID1/Wnt/β-catenin/c-MYC transcriptionally activates *G6PD*	(60)
		PTEN/Tcl1/hnRNPK regulates pre-mRNA splicing and activity of G6PD	(61)
		BAG3 inhibits dimerization and activity of G6PD	(62)
	Colorectal cancer	YY1 transcriptionally activates *G6PD*	(63)
		P53 inactivates G6PD	(64)
	Leukemia	mTORC1/SREBP1 transcriptionally activates *G6PD*	(65, 66)
		SIRT2 deacetylates G6PD at K403 which enhances its activity	(67)
	Breast cancer	NSD2 increases the level of H3K36me2 at the promoter of *G6PD* and enhances its expression	(68)
	Prostate cancer	TRIM21 promotes ubiquitination of G6PD	(69)
	Cervical cancer	Plk1 phosphorylates G6PD at T406 and T466 which promotes its active dimer formation	(70)
6PGD	Lung cancer	Nrf2 transcriptionally activates *6PGD*	(58)
		YTHDF2 binds to the m6A modification site of 6PGD mRNA and promotes its translation	(71)
		DLAT and ACAT2 acetylate 6PGD at K76 and K294 which enhances its activity, while HDAC4 deacetylates both sites	(72)
	Brain cancer	EGFR promotes phosphorylation of 6PGD at Y481 by Fyn which enhances its activity	(73)
RPI/RPE	Pancreatic cancer	Kras ^G12D^ transcriptionally activates *RPI/RPE*	(74)
TKT	Lung cancer	Nrf2 transcriptionally activates *TKT*	(58)
	Liver cancer	BACH1 transcriptionally represses *TKT*Nrf2 transcriptionally activates *TKT*	(75)
	Breast cancer	PFKFB4/SRC-3-ATF4 transcriptionally activates *TKT*	(76)
	Leukemia	BCR-ABL/HIF-1α transcriptionally activates *TKT*	(77)
	Pancreatic cancer	MUC1/HIF-1α transcriptionally activates *TKT*	(78)
TALDO	Lung cancer	Nrf2 transcriptionally activates *TALDO*	(58)

### Enzymes in the Oxidative PPP

**Figure 4:**
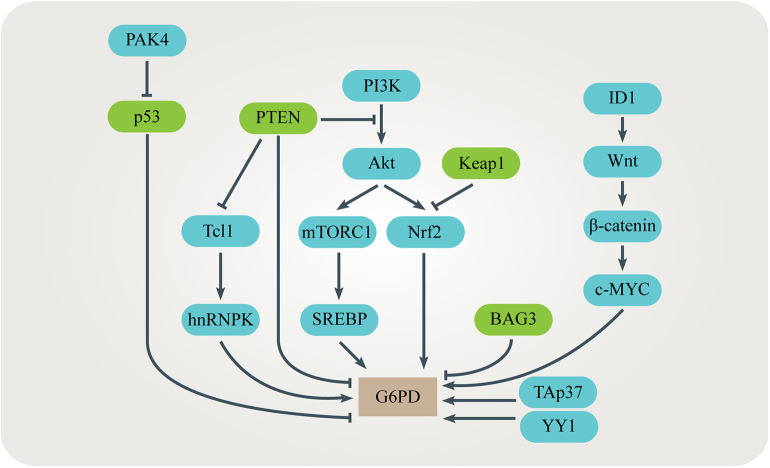
Regulation of G6PD in cancers. Several signaling pathways have been identified to be responsible for promoting G6PD expression or activity in cancer cells. These signaling pathways interact with each other, adding complexity to the regulation of G6PD.

Up-regulation of the **G6PD** level or activity is often observed in many kinds of cancer (79–86). Several signaling pathways have been identified to be responsible for promoting G6PD expression or activity in cancer cells (Figure 4). TP53-induced glycolysis and apoptosis regulator (TIGAR) enhances the PPP flux and biosynthesis (87). The tumor suppressor p53 directly binds to G6PD and prevents formation of the active G6PD dimer whereas the mutant p53 fails to inhibit G6PD in cancer cells (64). The p21-activated kinase 4 (PAK4) regulates G6PD activity by promoting p53 ubiquitination (81). Bcl-2 associated athanogene 3 (BAG3) inhibits dimerization and activity of G6PD (62). TAp73, a member of the p53 family which is often overexpressed in cancers, supports tumor growth by inducing G6PD expression (56, 57). Nuclear factor E2-related factor 2 (NRF2) is a transcription factor regulated by oxidative stress. When the PI3K/Akt signaling pathway is activated, NRF2 directly increases G6PD, 6PGD, TKT, and TALDO expression to enhance metabolic activities and promote cancer cell growth (58). The mammalian target of rapamycin complex 1 (mTORC1) stimulates the oxidative branch of the PPP by enhancing the sterol regulatory element-binding protein (SREBP)-dependent transcription of G6PD (65, 66). Inhibitor of differentiation and DNA binding-1 (ID1) regulates c-MYC through Wnt/β-catenin pathway activation to promote G6PD transcription and activate the PPP (60). Phosphatase and tensin homolog (PTEN) prevents G6PD from activation *via* the PTEN/Tcl1/hnRNPK/G6PD axis (61). Transcription factor yin yang 1 (YY1) regulates G6PD transcriptional activity by directly binding to the *G6PD* promoter (63). Histone H3K36 methyltransferase NSD2 methylates H3K36me2 at the *G6PD* promoter to up-regulate its expression (68). In addition, some post-translational modifications such as phosphorylation, acetylation, O-GlcNAcylation and ubiquitination affect the activity of G6PD (59, 67, 69, 70).

Activated G6PD increases flux through the oxidative branch of PPP. Up-regulation of the PPP flux provides cells with R5P for nucleotide biosynthesis, as well as NADPH for biosynthesis and maintaining redox homeostasis (59, 67, 70). The level of G6PD often negatively correlates to the prognosis of cancer patients (61). Suppression of G6PD induces cellular senescence in hepatocellular carcinoma (HCC) cells and leads to intracellular oxidative stress, making cancer cells sensitive to chemotherapy (60, 61). Interestingly, elevated G6PD is not observed in liver cirrhosis which is a main cause of liver cancer, indicating that G6PD might play an important role in promoting malignant transformation (88).

**6PGL** is found to be associated with shorter overall survival in breast cancer patients with bone metastases (89). However, the role of 6PGL in cancer remains to be elucidated.

Elevated **6PGD** and its product Ru5P inhibit AMPK activation by disrupting the active LKB1 complex, which promotes lipogenesis by abolishing AMPK-dependent acetyl-CoA carboxylase 1 (ACC1) phosphorylation and inactivation (90). 3-phosphoglycerate (3-PG), the intermediate product of glycolysis, inhibits 6PGD enzyme activity. Therefore, elevated glycolytic enzyme phosphoglycerate mutase 1 (PGAM1) keeps its substrate 3-PG to a relatively low level and promotes the PPP in cancer cells (91). In addition to its function as a metabolic enzyme, 6PGD also regulates cell metastasis by promoting phosphorylation of c-Met (92). 6PGD promotes the formation of distant metastatic subclones in pancreatic ductal adenocarcinoma (PDAC) by regulating epigenetic reprogramming. 6PGD inhibitor 6-aminonicotinamide (6AN) selectively and quantitatively reverses several reprogrammed chromatin modifications and blocks the tumorigenic potential of distant metastasis (93). How 6PGD and the PPP regulate epigenetic programs requires further investigation.

Elevated 6PGD expression and/or enzyme activity is also found in liver cancer, cervical cancer, thyroid cancer, breast cancer, ovarian cancer, non-small cell lung cancer (NSCLC) and leukemia (72, 94–99). In lung cancer, YTH domain family 2 (YTHDF2) promotes 6PGD mRNA translation by directly binding to the m6A modification site, leading to increased oxidative PPP flux (71). Post-translational modification of 6PGD is important for cancer cell proliferation and tumor growth. Acetylation of 6PGD plays a key role in coordinating redox homeostasis, lipogenesis and glycolysis (72). In glioma, epidermal growth factor receptor (EGFR) activation increases 6PGD phosphorylation and activation to promote DNA synthesis and resistance to radiation (73). Patients with lower levels of 6PGD Y481 phosphorylation have longer median survival time (73). Aberrant expression of 6PGD can accelerate cancer cell proliferation and induce resistance to chemical or radical therapy (72, 94–99). All these findings suggest that inhibiting the expression or activity of 6PGD might be a promising therapeutic strategy for cancer.

### Enzymes in the Non-oxidative PPP

**RPI** and **RPE** expression is induced by the oncogenic *Kras* mutation, which is critical for the initiation of PDAC. Kras^G12D^ regulates the non-oxidative but not oxidative PPP to provide cancer cells with sufficient R5P for nucleotide biosynthesis (74). RPI mRNA and protein levels are elevated in HCC. RPI promotes tumor growth and colony formation by negatively modulating protein phosphatase 2A (PP2A) to activate extracellular signal-regulated kinase (ERK) signaling pathways (100). In zebrafish, overexpression of RPI contributes to fatty liver, liver cirrhosis and cell proliferation (101). Therefore, whether and how RPI plays an important role in HCC development is worthy of further study. Furthermore, RPI increases the stability of β-catenin and promotes colorectal tumorigenesis by inducing Wnt target genes such as *Cyclin D1* in zebrafish (102). High level of RPI is reported to predict negative clinical outcomes of colorectal cancer patients. Moreover, miRNA-124 decreases glucose metabolism and cell growth in colorectal cancer by down-regulating RPI (103).

The expression of **TKT** is elevated in many types of cancer (75–78, 83, 104). BTB and CNC homolog 1 (BACH1) and kelch-like ECH-associated protein 1 (KEAP1) negatively regulate TKT expression while NRF2 positively regulates it (75). 6-phosphofructo-2-kinase/fructose-2, 6-bisphosphatase 4 (PFKFB4) phosphorylates steroid receptor coactivator-3 (SRC-3) at S857 which increases TKT expression (94). Hypoxia-inducible factor-1α (HIF-1α) induced in leukemia and pancreatic cancer enhances the non-oxidative arm of the PPP by promoting TKT activity, resulting in resistance to chemotherapy (77, 78). In addition to TKT, its two homologs, transketolase like-1 (TKTL1) and transketolase like-2 (TKTL2) are also found in human beings (105). TKT and TKTL1 rather than TKTL2 are essential for promoting cell growth and reducing oxidative stress in cancer cells (106, 107).

Patients with pancreatic cancer have higher levels of serum fructose which induces TKT expression to drive nucleic acid synthesis in cancer cells (104). TKT can maintain redox hemostasis by regulating the level of NADPH in liver cancer cells (75). Inhibition of TKT leads to increased ROS production and decreased glycolytic flux. Despite the accumulation of R5P, knockdown of TKT suppresses tumor growth and sensitizes cancer cells to chemotherapy (75). Recent work suggests that TKT promotes genome instability by regulating nucleotide biosynthesis during liver injury and cancer initiation (108). In addition, TKT can regulate cell cycle and promote the viability and proliferation of cancer cells independent of its enzyme activity. TKT interacting with EGFR and MAPK3 might be the underlying mechanism (109).

**TALDO** is highly expressed in gastric adenocarcinoma and HCC (110, 111). Moreover, higher TALDO expression often indicates poorer clinical outcomes and more resistance to trastuzumab therapy in breast cancer. When human epidermal growth factor receptor 2 (HER2) signaling is inhibited, breast cancer cells rely on the non-oxidative arm of the PPP to replenish the oxidative arm. Combined with HER2 inhibition, TALDO knockdown can exacerbate the reduction of NADPH and promote cell death (112). Surprisingly, TALDO can protect against cancer initiation. Loss of TALDO reduces GSH and diminishes β-catenin phosphorylation and Fas-dependent apoptosis, promoting hepatocarcinogenesis in mouse models (113).

## Could PPP be a Target For T2Dm and Cancer?

The major contributor to cytosolic NADPH is the PPP (114). NADPH, a key intracellular reductant, is required for glutathione system and other ROS scavengers to maintain the redox homeostasis (115). High ROS levels not only damage DNA, proteins and lipids to induce genome instability and activate NF-κB, PI3K, HIF-1α, and MAPK which contributes to carcinogenesis (116), but also result in T2DM (117, 118). Therefore, the PPP serves as an ideal target for regulating the redox homeostasis in metabolic diseases and cancer.

The PPP regulates insulin secretion. When insulin or insulin-like growth factors bind insulin receptor (IR) and insulin-like growth factor-I receptor (IGF-IR), many downstream signaling pathways including the Ras/Raf/Mek/Erk pathway and the PI3K/Akt/mTOR pathway are activated to drive cell growth and proliferation. Insulin promoting the growth and proliferation of cells is one of the mechanisms underlying increased cancer risk in obese and diabetic patients (119, 120). Chronic inflammation is a well-known hallmark of cancer and insulin resistance. Obesity-related inflammation is believed to create a microenvironment contributing to the initiation and progression of cancer (121). In turn, cancer cells secrete cytokines to recruit macrophages, leading to cancer-related inflammation, which plays an important role in cancer cell migration and invasion (122, 123). Therefore, targeting the PPP to block the M1 macrophage function is a possible strategy for both T2DM and cancer.

## Summary

The PPP plays a critical role in type 2 diabetes and cancer. Being the major source for NADPH, the PPP serves an ideal target for regulating the redox homeostasis in metabolic diseases and cancer. In addition, the intermediate R5P in the PPP is a precursor for nucleotide biosynthesis, which is essential for DNA replication and DNA damage repair. G6PD and 6PGD are the two enzymes in the PPP which catalyze the reactions to produce NADPH. Although TKT in the non-oxidative PPP does not directly catalyze the formation of NADPH, recent study has revealed its role in regulating cellular NADPH and R5P levels by balancing the flux between glycolysis and the PPP. Therefore, G6PD, 6PGD, and TKT are promising targets in the PPP for prevention and treatment of metabolic diseases and cancer.

## Author Contributions

XT designed and revised the manuscript. TG, JY, YW, SZ, and YL wrote the manuscript and made the figures.

## Conflict of Interest

The authors declare that the research was conducted in the absence of any commercial or financial relationships that could be construed as a potential conflict of interest. The handling editor is currently co-organizing a Research Topic with one of the authors XT, and confirms the absence of any other collaboration.
